# Estimation of the Carbon Footprint Associated With Attendees of the American Psychiatric Association Annual Meeting

**DOI:** 10.1001/jamanetworkopen.2020.35641

**Published:** 2021-01-28

**Authors:** Joshua R. Wortzel, Alec Stashevsky, Jeremy D. Wortzel, Beth Mark, Janet Lewis, Elizabeth Haase

**Affiliations:** 1 Group for the Advancement of Psychiatry, Climate Committee; 2 Department of Psychiatry, Strong Memorial Hospital, University of Rochester Medical Center Complex, Rochester, New York; 3 University of Pennsylvania School of Medicine, Philadelphia; 4 University of Pennsylvania Counseling and Psychological Services, Philadelphia; 5 Department of Psychiatry and Behavioral Sciences, University of Nevada School of Medicine, Reno

## Abstract

This cross-sectional study uses data from the 2018 and 2019 American Psychiatric Association (APA) annual meetings to assess the carbon dioxide equivalent emissions associated with the conferences and how they may be reduced.

## Introduction

The health care system produces 8% of US greenhouse gas emissions.^1^ International medical conferences contribute to these emissions substantially; 1 conference alone can produce the carbon dioxide equivalent (CO_2_*e*) emissions of an entire city in a single week.^2^ Virtual conferences necessitated by the coronavirus disease 2019 (COVID-19) pandemic have been associated with reduced emissions of up to 99.97%,^3^ and it is estimated that holding conferences biennially in accessible locations and increasing virtual presentations may be associated with reductions in emissions of 90%.^2^ The American Psychiatric Association (APA) has made addressing the effects of climate change on mental health one of its priorities,^4^ yet it holds one of the largest annual psychiatric conferences in the world. We calculated the carbon footprint associated with the 2018 and 2019 APA annual meetings and assessed how it can be optimally reduced.

## Methods

For this cross-sectional study, we obtained cities and countries of origin data for deidentified attendees of the 2018 APA Annual Meeting (May 5-9; n = 16 620) and the 2019 APA Annual Meeting (May 18-22; n = 13 335) from the APA. The Research Subjects Review Board at the University of Rochester determined that this research does not involve human participants as defined by the US Department of Health and Human Services and Food and Drug Administration regulations. This study followed the Strengthening the Reporting of Observational Studies in Epidemiology (STROBE) reporting guideline.

We identified likely transportation modes and departure airports for each attendee based on their geodesic distance from the meetings (ie, drivers, ≤400 km; flyers, >400 km). Driving emissions were estimated using the Environmental Protection Agency’s guidelines on passenger vehicles.^5^ Flying emissions were estimated using Flight Emissions API (GoClimate), which uses a radiative forcing index of 2—a conservative estimate.^6^ We explored carbon emissions for theoretical conferences if the 2018 and 2019 conference attendees had traveled to the past 40 APA meeting locations. In addition, we applied a geometric minimization algorithm to identify optimal meeting locations without geographic constraints. All analyses were performed using R, version 3.6.3 (R Project for Statistical Computing).

## Results

The 2018 New York City and 2019 San Francisco APA annual meetings were estimated to have produced 19 819 (1.19 per capita) and 21 456 (1.61 per capita) metric tons of CO_2_*e* emissions, respectively. For both meeting populations, theoretical conferences held in the western US and Hawaii were associated with estimated increases in carbon footprints by 21% to 164% compared with locations in the northeastern US (Table). The geometric minimization analysis corroborated that northeastern US locations were associated with optimized APA meeting emissions in worldwide location comparisons. Variations in CO_2_*e* emissions across locations were associated with the proportion of attendees within driving distance of locations. This proportion was minimized for conferences in the northeastern US because 36% to 55% of US attendees were from this region (Figure). Estimated emissions were also minimized in Northeastern US locations for international attendees, who were predominantly from Europe.

**Table.** Estimated CO_2_*e* Emissions Associated With APA Annual Meetings at Actual and Theoretical Meeting Locations^a^

Emissions rank	Location	Total CO_2_*e* emissions, metric ton	Per capita CO_2_*e* emissions, metric ton	Difference between actual and theoretical meeting emissions, %
**2018 New York City APA Annual Meeting**				
1^b^	New York City	19 819	1.19	NA
2	Philadelphia	19 965	1.20	1
3	Washington DC	20 304	1.22	2
4	Toronto	21 276	1.28	7
5	Montreal	21 597	1.30	9
6	Chicago	22 256	1.34	12
7	Atlanta	23 069	1.39	16
8	New Orleans	25 170	1.51	27
9	Dallas	25 678	1.55	30
10	Miami	25 898	1.56	31
11	Los Angeles	31 761	1.91	60
12	San Diego	31 768	1.91	60
13	San Francisco	32 708	1.97	65
14	Honolulu	52 415	3.15	164
**2019 San Francisco APA Annual Meeting**				
1	Washington DC	16 431	1.23	−23
2	Philadelphia	16 475	1.24	−23
3	Chicago	16 552	1.24	−23
4	New York City	16 555	1.24	−23
5	Toronto	16 741	1.26	−22
6	Atlanta	17 433	1.31	−19
7	Montreal	17 521	1.31	−18
8	Dallas	18 234	1.37	−15
9	New Orleans	18 473	1.39	−14
10	Miami	20 023	1.50	−7
11	Los Angeles	21 068	1.58	−2
12	San Diego	21 108	1.58	−2
13^b^	San Francisco	21 456	1.61	NA
14	Honolulu	37 564	2.82	75

Abbreviations: APA, American Psychiatric Association; CO_2_*e*, carbon dioxide equivalent; DC, District of Columbia; NA, not applicable.

^a^ Actual and theoretical conference CO_2_*e* emissions were calculated if attendees of the 2018 New York City and 2019 San Francisco meetings traveled to the locations of the past 40 APA annual meetings. Locations are ranked from the lowest to highest estimated emissions.

^b^ Estimated carbon footprints for conference populations in their actual locations.

**Figure.**
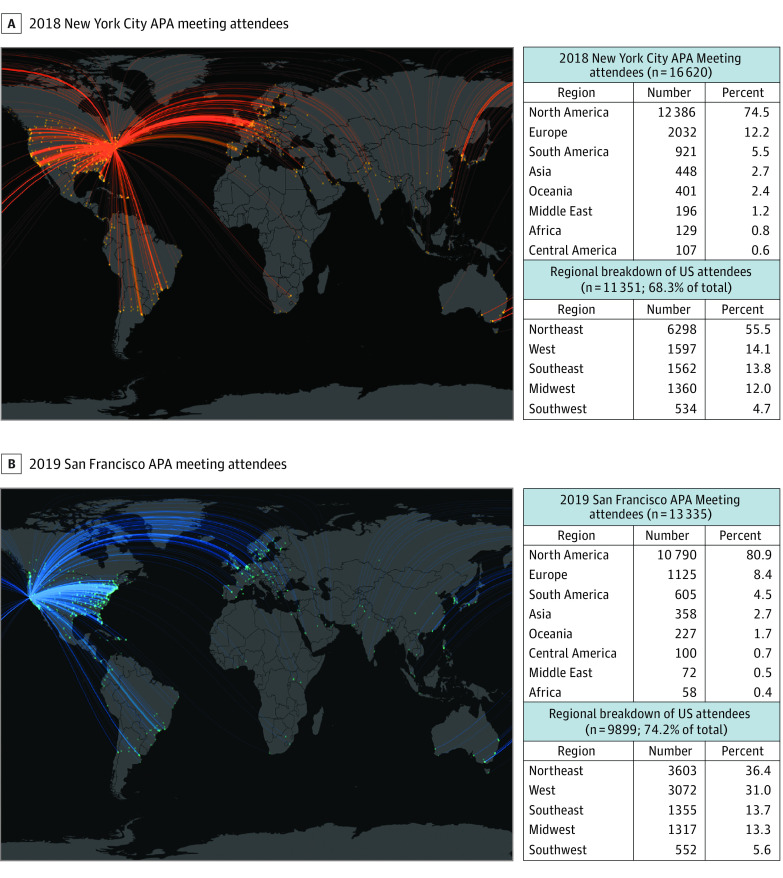
Places of Origin and Geodesic Distances of American Psychiatric Association (APA) Annual Meeting Attendees The size of each point is proportional to the number of attendees departing from that location. The intensity of the arcs is proportional to the number of attendees traveling that geodesic distance to conference locations.

## Discussion

The estimated carbon emissions associated with the APA annual meetings were significant and could vary 3-fold by conference location. Results of this study suggest that the APA saved the estimated equivalent of burning 500 acres of dense forest or 22 million pounds of coal by holding the 2020 conference virtually. These estimates are likely less than the actual carbon emissions saved. A limitation of this study was the use of geodesic distances to approximate travel routes, which are less circuitous than the actual routes attendees would have taken. The intent of this analysis was not to encourage the elimination of in-person conferences. Attending professional meetings is critical for socialization, networking, and learning that leads to advancement in clinical practice, research, and policy. These meetings also provide large sources of income for the APA. There is an ethical imperative, however, to reduce the significant health and environmental damage caused by conference travel. Optimizing conference location alone may be associated with achieving the emissions reductions targeted by the United Nations Paris Agreement. Creative workarounds, such as prorated registration costs for attendees who must fly to emissions-optimized locations, could make this strategy more equitable. Use of intermittent virtual formats may be associated with further reductions in the carbon footprint associated with the APA meetings. All of these solutions exemplify a needed shift in the mindset of the medical community; sustainability does not have to be a zero-sum proposition in which the needs of clinicians, patients, and the planet are at odds. Instead, a more creative and intentional approach can be taken that meets our responsibility to do no harm as we innovate for new planetary realities.
